# Inactivation of Retinoblastoma Protein (Rb1) in the Oocyte: Evidence That Dysregulated Follicle Growth Drives Ovarian Teratoma Formation in Mice

**DOI:** 10.1371/journal.pgen.1005355

**Published:** 2015-07-15

**Authors:** Qi-En Yang, So I. Nagaoka, Ivy Gwost, Patricia A. Hunt, Jon M. Oatley

**Affiliations:** Center for Reproductive Biology, School of Molecular Biosciences, College of Veterinary Medicine, Washington State University, Pullman, Washington, United States of America; Syracuse University, UNITED STATES

## Abstract

The origin of most ovarian tumors is undefined. Here, we report development of a novel mouse model in which conditional inactivation of the tumor suppressor gene *Rb1* in oocytes leads to the formation of ovarian teratomas (OTs). While parthenogenetically activated ooctyes are a known source of OT in some mutant mouse models, enhanced parthenogenetic propensity *in vitro* was not observed for Rb1-deficient oocytes. Further analyses revealed that follicle recruitment and growth is disrupted in ovaries of mice with conditional inactivation of *Rb1*, leading to abnormal accumulation of secondary/preantral follicles. These findings underpin the concept that miscues between the germ cell and somatic compartments cause premature oocyte activation and the formation of OTs. Furthermore, these results suggest that defects in folliculogenesis and a permissive genetic background are sufficient to drive OT development, even in the absence of enhanced parthenogenetic activation. Thus, we have discovered a novel role of Rb1 in regulating the entry of primordial oocytes into the pool of growing follicles and signaling between the oocyte and granulosa cells during the protracted process of oocyte growth. Our findings, coupled with data from studies of other OT models, suggest that defects in the coordinated regulation between growth of the oocyte and somatic components in follicles are an underlying cause of OT formation.

## Author Summary

Ovarian teratomas (OTs) are the most frequent germ cell tumors in adult women, but their origin and molecular etiology remains poorly defined. We found that conditional deletion of the tumor suppressor *Rb1* in the oocyte leads to OT development in young adult female mice. Further analysis revealed disturbances in both recruitment and growth of follicles. Although oocytes from mutants did not exhibit an enhanced propensity for parthenogenetic activation–a proposed source of OTs–premature meiotic resumption was evident in oocytes of immature follicles. These findings, together with data from previous studies, suggest that a defect in oocyte-somatic cell communication leading to an uncoupling of coordinated growth and ultimately impaired sustainment of meiotic arrest is sufficient to drive OT development.

## Introduction

Ovarian tumors are a major source of cancer in women of all ages and can arise from both germ cells and somatic cells. Germ cell tumors account for approximately 5 to 20% of ovarian cancers and mainly affect young women [1,2]. These neoplasias are classified in many ways including dysgerminoma, yolk sac tumor, embryonal carcinoma, polyembryoma, ovarian teratoma (OT), and gonadoblastoma [3]. Mature, immature and monodermal OT subtypes constitute 95% of germ cell tumors in adult women [4,5]. The majority are benign, however, immature and cystic mature teratomas are capable of malignant transformation [6,7]. Currently, the origins and molecular etiology of ovarian germ cell tumorigenesis are poorly understood.

In mammals, germ cell fate is specified during embryogenesis in a subset of cells termed primordial germ cells (PGCs) that serve as precursors of both male and female germ cell lineages in postnatal life [8]. Following sex determination, PGCs enter mitotic quiescence in the fetal testis and these prospermatogonia are the precursors of the stem and progenitor spermatogonia in the postnatal testis [9,10]. In females, PGCs differentiate into oogonia, initiate meiosis, and arrest during meiotic prophase prior to birth [11,12]. During late fetal or early postnatal development, meiotically arrested oocytes become surrounded by somatic cells, forming primordial follicles. This pool of primordial follicles is a non-renewing reservoir of oocytes that is subject to selection and growth from puberty until the age of reproductive senescence [13]. PGCs possess the plasticity to contribute to the germ cell lineage and form pluripotent cells *in vitro*, but, under normal conditions, pluripotency is lost upon the initiation of meiosis [14,15].

In humans, defects in the transition of PGCs to meiotically arrested oocytes, including aberrant mitosis and failure to maintain meiotic arrest predispose oocytes to tumorigenic transformation [6,16,17]. Genetic screening of ovarian cancer patients has revealed an association between OT development and mutations in genes that encode tumor suppressors and oncogenes including *c-Kit*, *Pten*, *Kras* and *p53* [18,19,20]. Mutational inactivation of the tumor suppressor retinoblastoma protein 1 (*Rb1*) gene has been detected in most types of human cancers, including human ovarian cancers and teratomas [19,21,22]. In addition, ovarian germ cell tumors have been reported to arise in some women with retinoblastoma disease, suggesting a potential tumor suppressor role of Rb1 in the ovary [23]. Despite these findings, the influence of Rb1 loss-of-function on female germ cell development and tumor formation remains unclear.

In mice, studies of the OT susceptible LT/Sv strain and several knockout or transgenic lines suggest that OTs originate from oocytes that fail to maintain meiotic arrest and undergo parthenogenetic activation [24,25,26,27]. However, parthenogenetic activation is not the sole inducer, indicating a complex etiology of the pathology [6,26,28]. In this study, we generated a mouse model with functional inactivation of the *Rb1* gene in the germline and observed a novel route to OT formation. Our results indicate that inactivation of Rb1 activity in the oocyte causes an uncoupling of coordinated growth with granulosa cells leading to premature oocyte activation and the formation of both classical and cystic teratomas.

## Results

### Fertility is reduced in *Rb1* germ cell conditional knockout female mice

**Fig 1:**
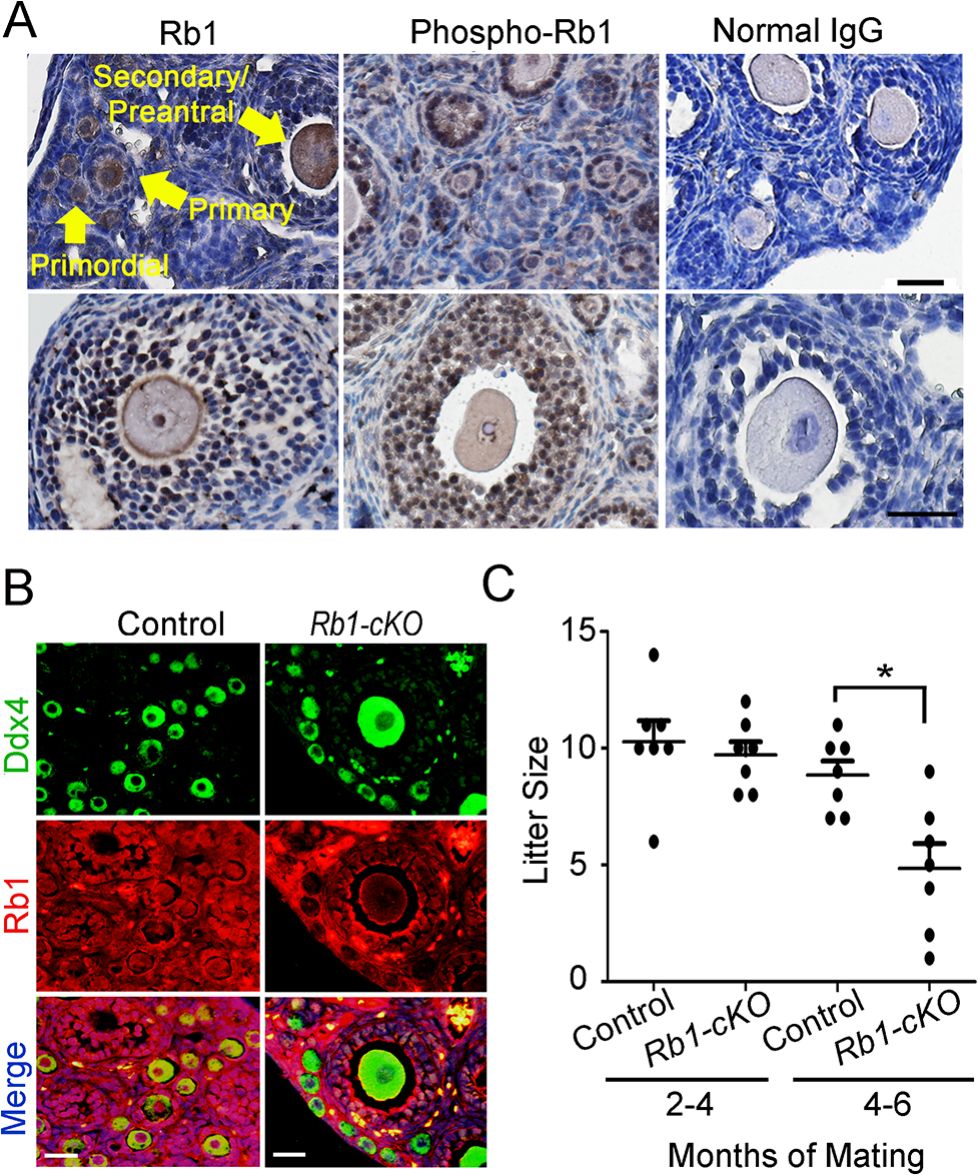
Impaired fertility of mice with conditional *Rb1* inactivation in oocytes. (A) Representative images of immunostaining with antibodies to Rb1 and phospho-Rb1 in ovarian cross-sections from wild-type adult mice. Normal IgG was used as a negative control. Arrows indicate different stages of developing follicles (i.e. primordial, primary, and secondary/preantral) and the scale bar = 50μm. (B) Representative images of co-immunofluorescence staining for Rb1 and the germ cell marker Ddx4 in ovarian cross-sections from adult control and *Rb1-cKO* mice. DNA was labeled with DAPI. Scale bar = 50μm. (C) Number of pups born from mating of control and *Rb1-cKO* females with wild-type males at 2–4 months and 4–6 months after pairing, n = 7 mice of each genotype and * denotes significantly different at P<0.05.

The tumor suppressor protein Rb1 is expressed in pre- and post-migratory PGCs of both sexes [29]. In the ovaries of juvenile and adult mice, Rb1 is detectable in both somatic and germ cells, although its role in the oocyte is undefined. Rb1 activity is controlled by phosphorylation, and growth factors can rapidly induce Rb1 phosphorylation at Ser780, Ser795 and Ser807/811 causing transient inactivation of function [30]. We found that phospho-Rb1 (Ser780) is abundantly detectable in granulosa cells and oocytes (Fig 1A and S1 Fig). Relative to total Rb1, phospho-Rb1 is high in granulosa cells and low in oocytes, indicating that its role as a cell cycle inhibitor is inactive in a majority of granulosa cells but active in growing oocytes. To assess its role functionally, mice with conditional inactivation of *Rb1* in the germline were generated by mating females homozygous for an *Rb1* floxed allele (*Rb1* ^*fl/fl*^) with young (<3 months of age) males carrying a *Ddx4*-*Cre* transgene. The resulting *Ddx4-Cre*; *Rb1* ^*fl/∆*^ (*Rb1-cKO*), *Ddx4-Cre;Rb1* ^*fl/+*^, *Ddx4-Cre;Rb1* ^*+/∆*^ and *Rb1* ^*fl/fl*^ females were used to examine the impact of homozygous or heterozygous inactivation of the *Rb1* gene on germ cell function and fertility. Co-immunostaining of cross-sections from ovaries of *Rb1-cKO* mice for the germ cell marker Ddx4 and Rb1 revealed that Rb1 protein was absent in oocytes of *Rb1-cKO* but present in somatic cells, consistent with specific inactivation in the germline (Fig 1B). To assess the fertility status of *Rb1-cKO* and control (*Rb1* ^*fl/fl*^) mice, females were paired with wild-type males beginning at 45 days of age and the number of pups born was recorded over a period of greater than 6 months. While the average number of pups was numerically lower for *Rb1-cKO* females compared to controls beginning at 3 months of pairing, the difference was not statistically significant (P>0.05). However, 6 months after pairing, the number of offspring born was significantly reduced by 40% for *Rb1-cKO* females compared to controls (Fig 1C).

### *Rb1-cKO* females develop unilateral OT

**Fig 2:**
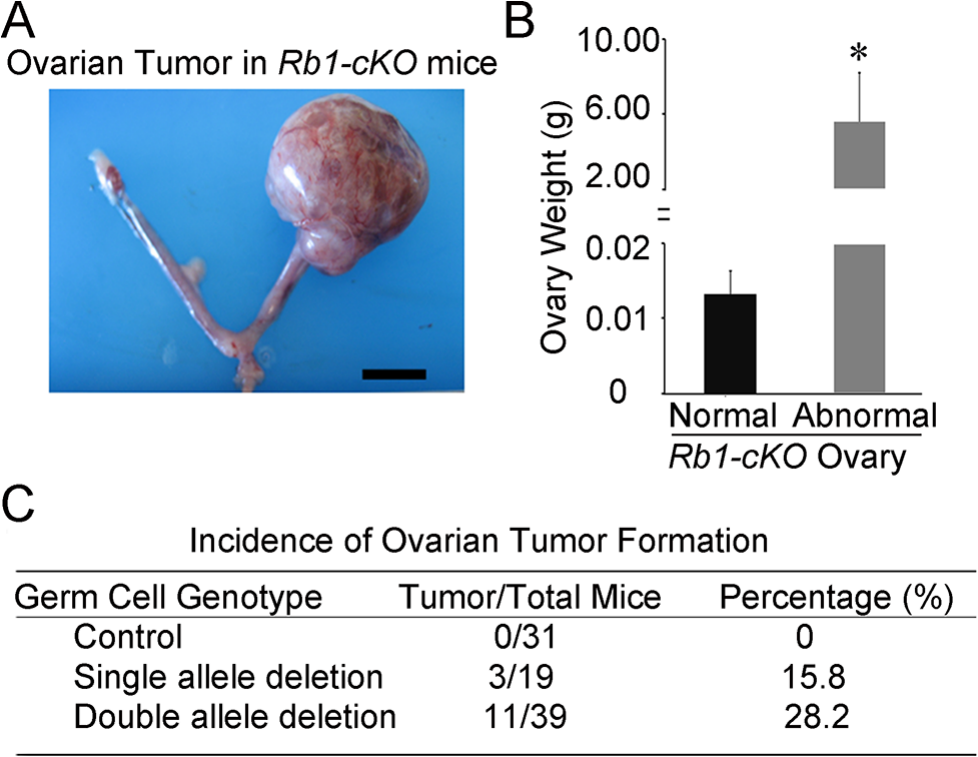
Ovarian tumor formation in mice with conditional *Rb1* inactivation in oocytes. (A) Representative image of the reproductive tract from an *Rb1-cKO* mouse. Scale bar = 1cm. (B) Average weight of normal and abnormal ovaries from *Rb1-cKO* mice. Data are mean±SEM and n = 4 mice of each genotype. *denotes significantly different at P<0.05. (C) Incidence of ovarian tumor formation at 3 months of age in control (*Rb1* ^*fl/fl*^, *Rb1* ^*fl/+*^), heterozygous *Rb1-cKO* (*Ddx4-Cre;Rb1* ^*fl/+*^ and *Ddx4-Cre;Rb1* ^*+/∆*^) and homozygous *Rb1-cKO* (*Ddx4-Cre;Rb1* ^*fl/∆*^ or *Rb1-cKO*) female mice.

**Fig 3:**
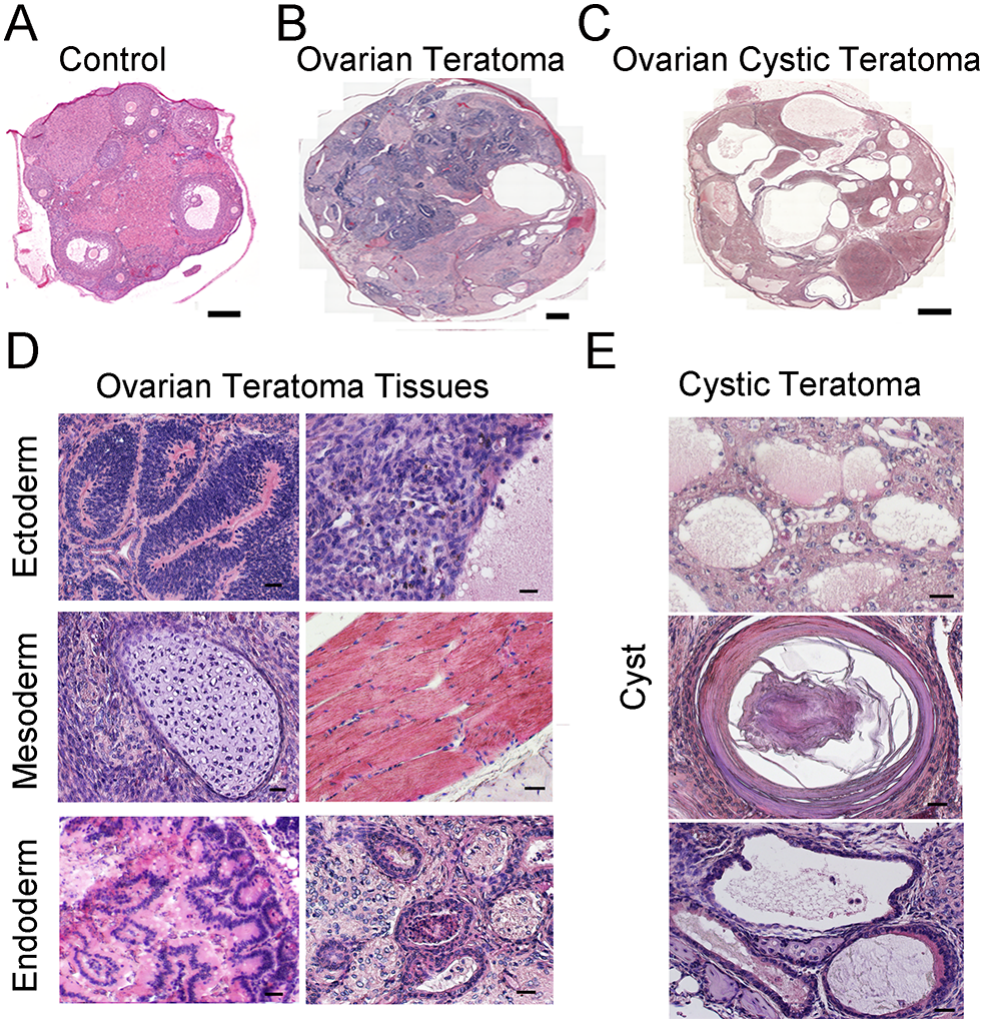
Formation of ovarian teratomas in mice with conditional *Rb1* inactivation in oocytes. (A-C) Representative images of hematoxylin and eosin (H&E) stained cross-sections from ovaries of control mice (A), *Rb1-cKO* mice with classical teratoma (B), and *Rb1-cKO* mice with cystic teratoma (C). Scale bars = 100μm (A) and 500μm (B and C). (D) Representative images of H&E stained cross-sections from an ovary with classical teratoma displaying endoderm, ectoderm and mesoderm-derived tissues. Scale bar = 50μm. (E) Representative images of H&E stained cross-sections from an ovary with cystic teratoma. Scale bar = 50μm.

To investigate the cause of reduced fertility in *Rb1-cKO* females, cross-sections of ovaries from 10 week old mice were examined. We found abnormal growths in the ovaries of *Rb1-cKO* mice but not controls (Fig 2A and S2A and S2B Fig). The average weight of abnormal ovaries was 5.8±2.5g (mean±SEM, n = 4), constituting 19.2% of the body weight for *Rb1-cKO* mice (Fig 2B). None of the control animals possessed the neoplasia (n = 31), indicating that genetic background did not influence spontaneous ovarian tumor formation. The abnormal ovarian phenotype was observed in 28.2% of homozygous and 15.8% of heterozygous *Rb1-cKO* mice (Fig 2C), and regardless of genotype, all ovarian abnormalities were unilateral and the contralateral ovary was of normal size. Results of a previous study suggests that expression of the *Ddx4-Cre* transgene can also occur in skin epithelium or globally in certain situations [31]. Thus, to confirm that the ovarian tumor phenotype arises due to *Rb1* inactivation in the germline, we conducted two experiments. First, we crossed *Ddx4-Cre* mice with *Flox-Stop-Rfp* reporter mice. In the ovaries of adult females, RFP signal was detectable in oocytes exclusively (S3 Fig). Second, we generated other *Rb1-cKO* females by mating *Rb1* ^*fl/∆*^ females and males carrying a *Blimp1-Cre* transgene that is active in PGCs [32]. In agreement with the data from *Ddx4-Cre*;*Rb1* ^*fl/∆*^ mice, ~30% of *Blimp1-Cre;Rb1* ^*fl/∆*^ females developed ovarian tumors (S4A and S4B Fig). All mice possessing ovarian growths died within 2 to 3 months after developing tumors likely due to intestinal obstruction and hemorrhagic infarction. We did not find signs of malignant transformation. Histological analyses confirmed that the neoplasia were OT, consisting of cell types from all three germ layers (Fig 3A–3E). In the early stage of development (2–4 months of age), classical teratomas consisting of endoderm (e.g. glandular epithelium), mesoderm (e.g. muscle tissue), and ectoderm (e.g. epidermis) derivatives were evident (Fig 3D). In older females (4–6 months of age), two different types of teratomas were observed. The first type contained tissue arising from the three germ layers, resembling classical teratomas (Fig 3B and 3D). The second type contained large cysts filled with a small area of epithelial and glandular tissues (Fig 3C and 3E). These cystic tumors resembled human cystic teratomas or dermoid cysts [33], although hair and other mature epidermal tissues were not observed. Interestingly, cells staining for the pluripotency markers Oct4, and Nanog were observed in cross-sections of OTs from *Rb1-cKO* mice at the early stage of development but not control counterparts (S5 Fig), suggesting that differentiated cells in the mature teratomas were derived from cells that regain pluripotency in the ovary.

### Follicle recruitment is abnormal in young *Rb1-cKO* animals

**Fig 4:**
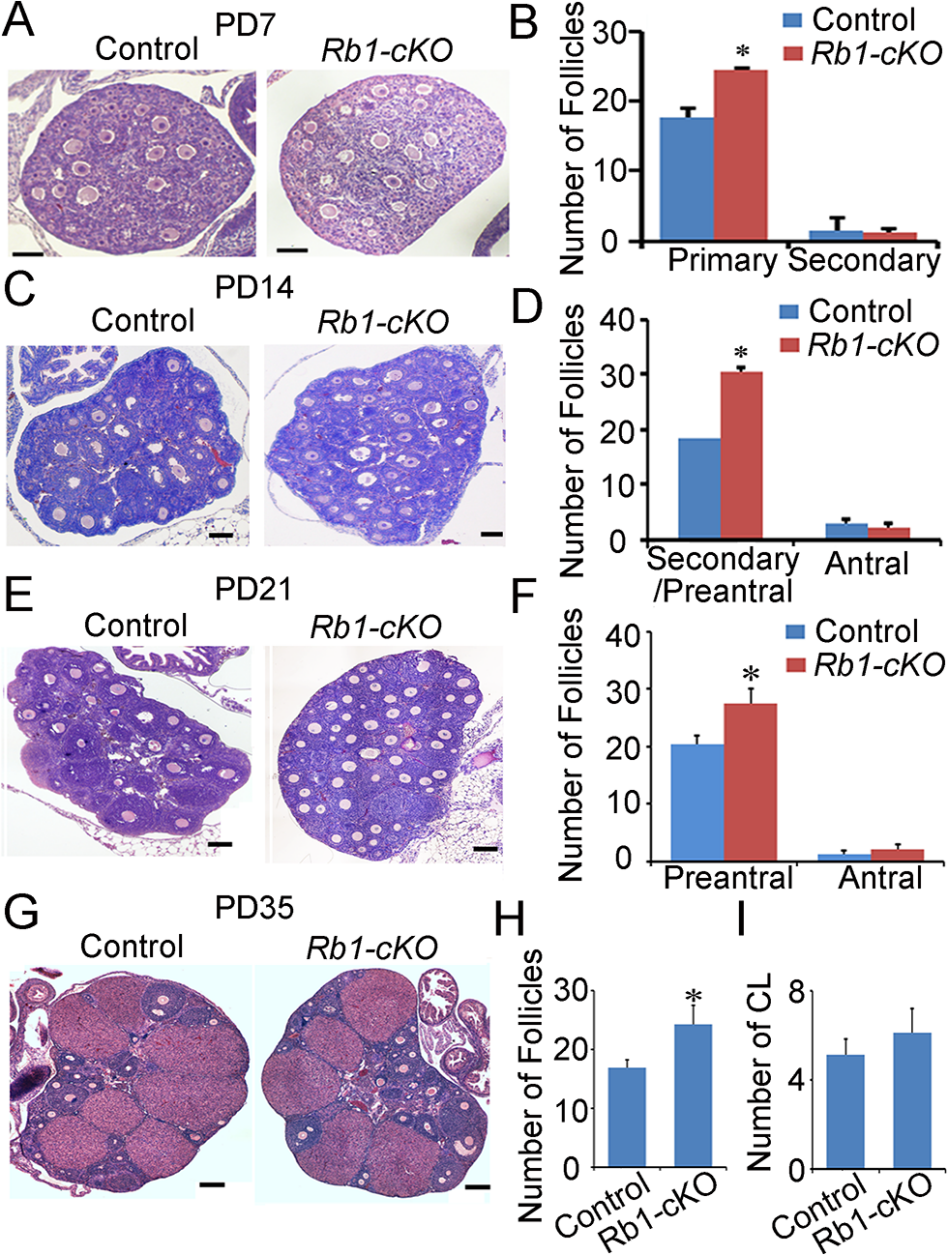
Aberrant follicle development in mice with conditional *Rb1* inactivation in oocytes. (A) Representative images of hematoxylin and eosin (H&E) stained cross-sections of ovaries from PD 7 control and *Rb1-cKO* females. (B) Quantitative comparison of the number of primary and secondary follicles at PD 7. (C) Representative images for H&E stained cross-sections from PD 14 control and *Rb1-cKO* ovaries. (D) Quantitative comparison of the number of secondary/preantral and antral follicles at PD 14. (E) Representative images of H&E stained cross-sections from PD 21 control and *Rb1-cKO* ovaries. (F) Quantitative comparison of the number of preantral and antral follicles at PD 21. (G) Representative images of H&E stained cross-sections from PD 35 control and *Rb1-cKO* ovaries. (H and I) Quantitative comparison of the number of antral follicles (H) and corpora lutea (CL; panel I). Data are mean±SEM of for at least 3 mice per group. Scale bars = 20μm for A, and 50μm for B, C, and D.

Considering the severe phenotype of mature female mice with oocyte-specific inactivation of *Rb1*, we became interested in determining the time of OT onset. To address this, cross-sections of ovaries from control and *Rb1-cKO* mice at postnatal days (PD) 7, 14, 21, 35, 150 and 180 were examined histologically. In mice, germ cell nest break down and formation of primordial follicles occurs during the first several days after birth. With the onset of puberty, cohorts of primordial follicles are recruited into the pool of growing oocytes each cycle, and the transition from primary follicle to mature preovulatory antral follicle takes approximately 18–24 days [34]. At each age, we quantified the number of primary, secondary/preantral and antral follicles in cross-sections of ovaries using criteria described previously [28]. Results of these analyses revealed a significant increase in the number of primary follicles in *Rb1-cKO* females at PD 7 by comparison with age matched controls (Fig 4A and 4B). At PD 14 and 21, preantral follicle numbers were greater in ovaries of *Rb1-cKO* mice (Fig 4D–4F). In addition, by PD 21 it was clear that, although the oocytes in the first wave of growing follicles had appropriately increased in size and were comparable to those in control ovaries, the growth of the somatic compartment was compromised in many follicles (Fig 4E). Notably, follicle number in ovaries of *Rb1-cKO* mice did not differ from controls at PD 2 (S6 Fig). These findings suggest that loss of Rb1 function in the oocyte has two distinct effects on folliculogenesis: first, it results in a slight increase in the number of follicles recruited in the first wave; second, and more importantly, it alters the kinetics of follicle growth, leading to the accumulation of abnormal secondary follicles in which oocyte growth appears to proceed normally despite the inability of the granulosa cell compartment to keep pace. Intriguingly, the number of antral follicles was not different between *Rb1-cKO* and control mice (Fig 4D and 4F), nor was there a difference in the number of corpa lutea at PD 35 (Fig 4H). A similar increase in preantral follicle number was evident in ovaries from animals at PD 45, 150, 180 and 240 (S7 Fig), suggesting that the folliculogenesis defects are not restricted to the first wave of follicles that initiate growth in the adolescent ovary. However, as in juvenile females, neither the number of antral follicles nor corpa lutea was different between *Rb1-cKO* and control mice, suggesting that elevated atresia counteracts the increase in number of oocytes ovulated by *Rb1-cKO* females.

### The frequency of parthenogenetic activation is not altered for *Rb1-null* oocytes

**Fig 5:**
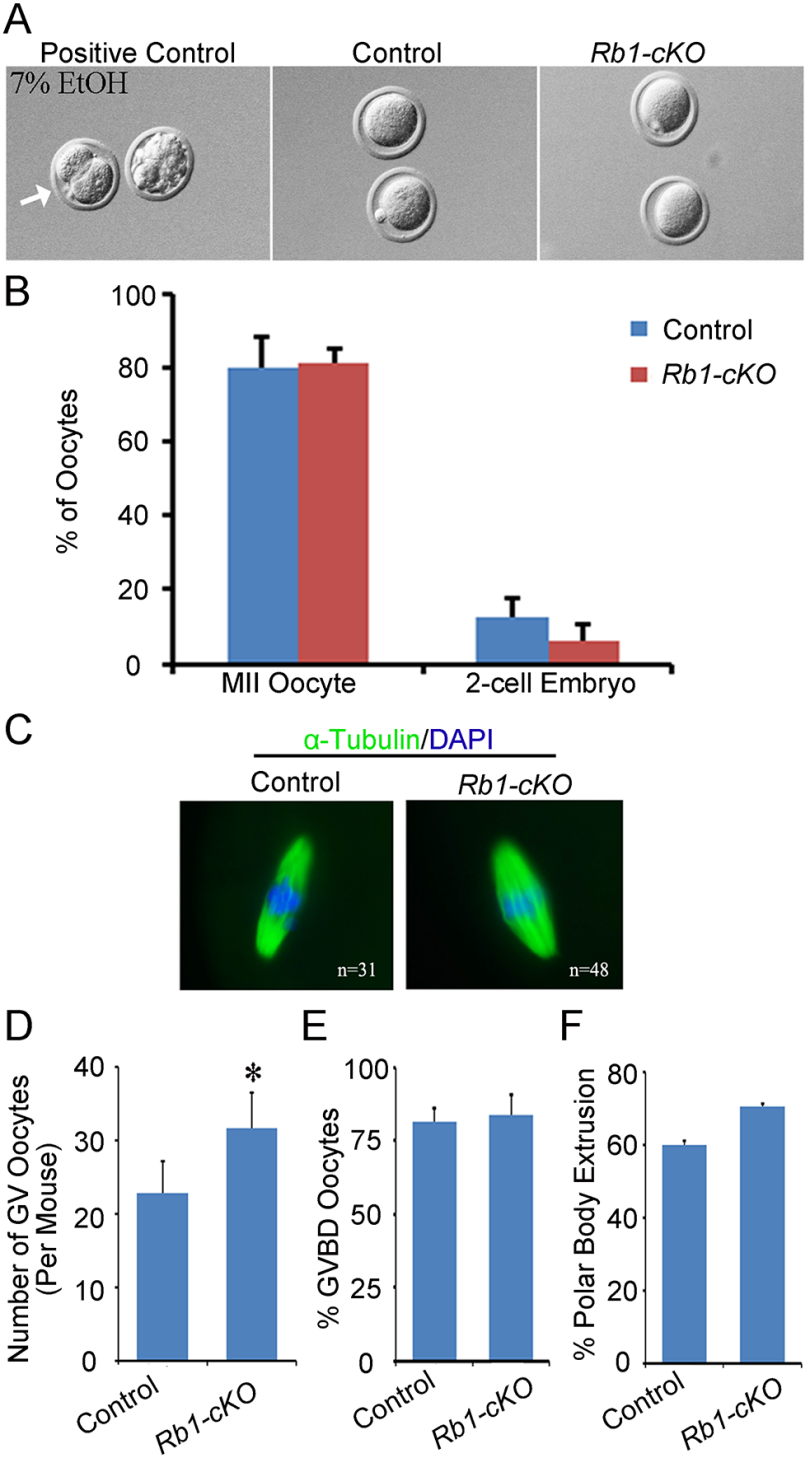
Propensity for parthenogenetic activation is not increased in *Rb1* deficient oocytes. (A) Representative bright field images of MII arrested oocytes from control and *Rb1-cKO* mice after 48 h of culture. Oocytes from control mice treated with 7% ethanol (EtOH) served as a positive control. (B) Quantitative comparison of the percent parthenogenetic activation (cleavage to 2-cell stage) after 48 h in culture for eggs from control and *Rb1-cKO* females. (C) Representative images of MII oocytes from control and *Rb1-cKO* mice immunostained for α-Tubulin and possessing a normal MII spindle (green) and chromosome alignment (blue). (D) Quantitative comparison of germinal vesicle (GV) stage oocytes recovered from control and *Rb1-cKO* mice 44–45 h after PMSG stimulation. (E and F) Quantitative comparison of the number of oocytes undergoing GV breakdown (GVBD) (E) and first polar body (PB) extrusion after 2–17 h in culture (F). Data represent mean±SEM for 3 mice of each genotype; *denotes significant difference at P<0.05.

Multiple lines of evidence suggest that the underlying cause of OT is parthenogenetic activation of oocytes in the ovary [26,35]. To explore whether a similar mechanism is the cause of OTs in *Rb1-cKO* mice, two different experimental approaches were undertaken. First, metaphase II arrested eggs were collected 12 h post hCG injection from the oviducts of superovulated control and *Rb1-cKO* female mice at PD 23. To assess the propensity for spontaneous activation and cleavage to the 2-cell stage, eggs were cultured for 48 h in either Waymouth’s MB752/1 or MEM media and percent activation scored as described previously [27]. Activation of control eggs with ethanol resulted in greater than 50% development to the 2-cell stage, demonstrating that culture conditions were sufficient to support parthenogenetic activation (Fig 5A). However, regardless of culture media, parthenogenetic activation of *Rb1* deficient oocytes was not enhanced *in vitro* (Fig 5B). The number of eggs that developed to the 2-cell stage in culture was not significantly increased in *Rb1-cKO* females in either Waymouth’s (6.4±4.0% in *Rb1-cKO* mice and 12.6±4.0% in control; P>0.05) or MEM culture medium (8.9±4.3% in *Rb1-cKO* mice and 13.3± 0.1% in controls; P>0.05). In a second set of studies, germinal vesicle (GV) stage oocytes were collected from stimulated ovaries 44–45 h post PMSG injection to assess meiotic progression. In agreement with the increased number of growing follicles from histological analyses of juvenile females, a greater number of GV stage oocytes was recovered from *Rb1-cKO* mice compared to controls (Fig 5D). Although the percentage of oocytes undergoing germinal vesicle breakdown (GVBD) was not different in control and *Rb1-cKO* mice (Fig 5E), the rate of polar body extrusion was slightly higher in *Rb1*-deficient oocytes (Fig 5F). Importantly, when MII eggs were cultured for an additional 24 h (Fig 5C), there was no evidence of spontaneous activation in either group. Overall, these findings demonstrate that inactivation of *Rb1* in oocytes does not increase the propensity for parthenogenetic activation *in vitro*, suggesting that impaired maintenance of meiotic arrest is not the driving force of teratoma formation.

### Teratomas are derived from oocytes within the ovaries of *Rb1-cKO* mice

**Fig 6:**
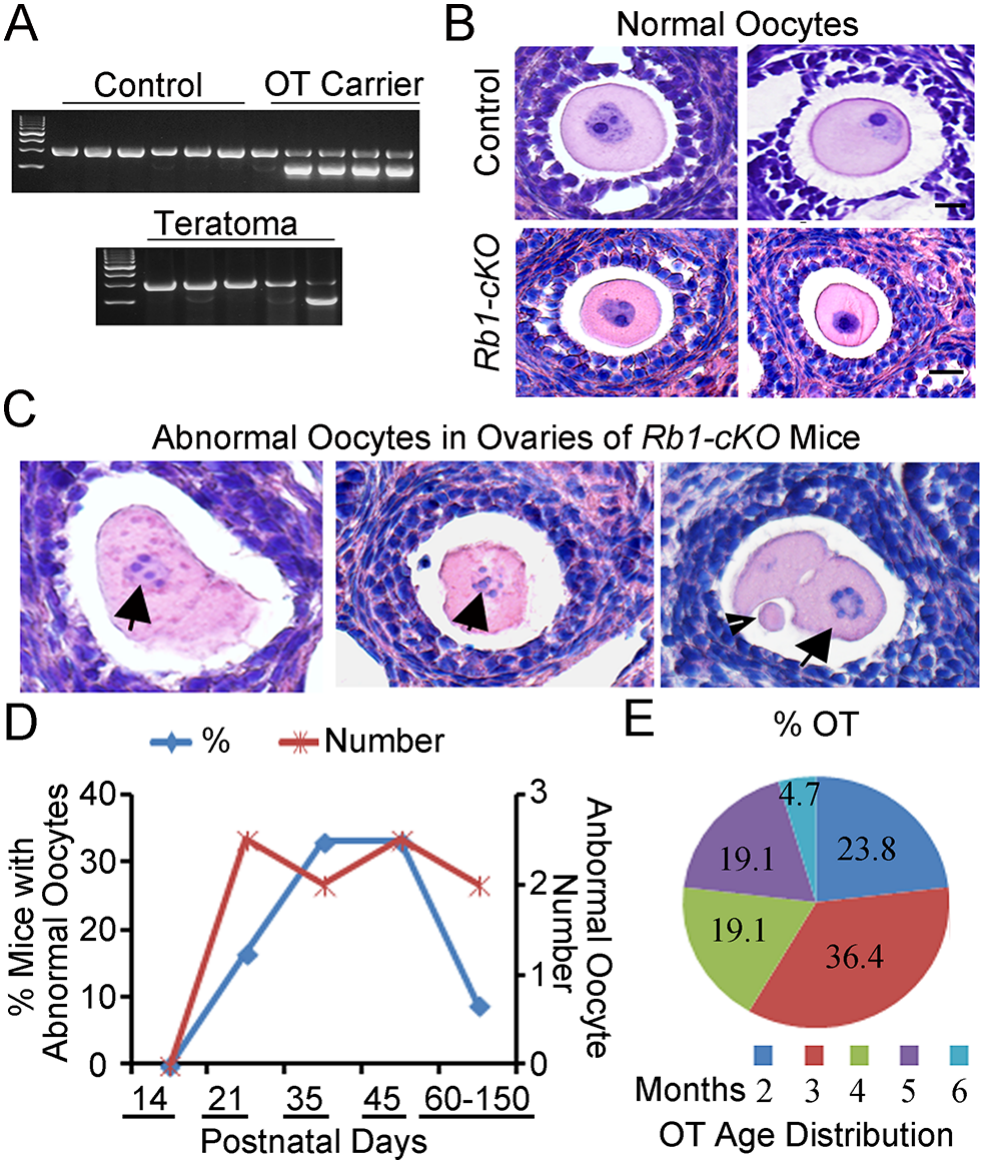
Premature resumption of meiosis in preantral follicles of mice with conditional *Rb1* inactivation in oocytes. (A) Representative images of agarose gels from genotypic analysis of teratomas and host somatic tissues for the *Ddx4-Cre* transgene. (B-C) Representative images of hematoxylin and eosin (H&E) stained cross-sections of normal follicles in ovaries from control and *Rb1-cKO* mice at PD 21 and 35 (B) and abnormal follicles from *Rb1-cKO* mice (C). Arrows indicate abnormal oocytes with condensed chromatin. Arrowhead indicates polar body in an apparent 2-cell embryo. (D) Percentage of *Rb1-cKO* mice at advancing postnatal ages (n = 6–7 mice at each age point and 33 mice total) that contained abnormal oocytes in ovarian cross-sections (blue line and left vertical axis) and the average number of abnormal oocytes observed per cross-section (red line and right vertical axis). (E) Age distribution of *Rb1-cKO* mice (n = 10) that developed ovarian teratomas (OT).

Recent studies in mice have demonstrated that oocyte activation leading to embryo formation in the ovary does not always produce teratomas [36]. Thus, parthenogenesis is not sufficient for teratoma formation. In humans, OTs are known to arise from multiple sources including, PGCs that fail to enter meiosis I during fetal development, and premature activation of fully formed oocytes following the completion of meiosis I or II [6,37] Because teratoma formation in *Rb1-cKO* mice was first detectable in 8 to 10-week old ovaries, sustainment of a PGC state seemed unlikely to be the underlying cause. We reasoned that, if PGCs were the source of teratomas, cells expressing hallmarks of pluripotency would be detectable in ovaries of young *Rb1-cKO* mice. However, neither expression of Nanog nor Oct4 which are classical markers of pluripotent cells was detectable in *Rb1*-deficient ovaries at PD 14, 21, or 35 (S8A Fig), providing further evidence that persistent PGCs are not the source of teratomas in *Rb1-cKO* mice. Further, Rb1 was successfully deleted in teratoma tissues (S8B Fig), and we found both *Ddx4-cre* transgene positive and negative teratomas, suggesting that teratomas arise from oocytes that have completed the first meiotic division (Fig 6A).

Because an increased propensity for parthenogenetic activation was not observed in oocytes of *Rb1-cKO* mice *in vitro*, we next evaluated oocytes *in vivo*. Examination of ovarian cross-sections from control and *Rb1-cKO* mice at PD 14, 21, and 35 demonstrated several significant aberrations. Although structures resembling developing embryos similar to those described in ovaries of *c-Mos* and *Cpeb* mutant mice [27,36] were not observed within the ovaries of any *Rb1-cKO* mice, our analysis revealed several significant aberrations (Fig 6B and 6C). Condensed chromatin was frequently evident in the oocytes of *Rb1-cKO* mice. In addition, rare follicles containing what appeared to be a 2-cell embryo with or without an evident polar body were observed (Fig 6C), suggesting that at least some oocytes had prematurely resumed and completed the first meiotic division. Interestingly, all abnormal oocytes were found in small preantral follicles and, notably, in follicles containing large oocytes (Fig 6C). Lastly, we examined the number of abnormal oocytes in cross-sections of ovaries from control and *Rb1-cKO* females. The outcomes revealed the presence of 0, 2.5±0.7, 2.0±0.1, 2.5±0.7, and 2.0±0.1, abnormal oocytes per cross-section in *Rb1-cKO* mice at PD 14, 21, 35, 45, and 60–150, respectively (Fig 6D). Abnormal oocytes were not observed in any cross-sections of ovaries from control mice examined (n = 10 mice and an age range of 21–180 days). Interestingly, the percentage of *Rb1-cKO* mice with abnormal oocytes was approximately 17% at PD 21 but increased to 33% at PD 35–45 and then decreased to only 10% at PD 180 (Fig 6D). Coincidently, we examined the age distribution of mice that developed OTs and discovered the majority of tumors formed between 2 and 5 months of age (Fig 6E). These observations suggest that mismatched kinetics of oocyte and granulosa cell growth is the major cause of OT formation in young *Rb1-cKO* mice. Multiple abnormal oocytes were evident in a given ovary of *Rb1-cKO* mice from PD 23 to 35; however, only rare cysts were present at the early stage of teratoma development, indicating not all activated oocytes became OTs. The lack of evidence for more advanced stages of embryogenesis in ovaries of *Rb1-cKO* mice may simply reflect the relative rarity of teratoma formation or more rapid disorganization for the developmental background of the strain.

## Discussion

Three different factors have been associated with OTs in mice; genetic background, meiotic abnormalities, and aberrations in follicle development. The first report of spontaneous OTs was in the LT/Sv strain [26,38]. In these mice, a meiotic abnormality causes arrest of a large number of oocytes at metaphase I, and the incidence of parthenogenetic activation is increased. Genetic studies demonstrated that teratoma formation is a polygenic trait in LT/Sv mice and that the propensity for parthenogenetic activation and tumor formation are enhanced by a single autosomal recessive gene derived from the C57BL/6 background [26]. OTs also have been reported in a number of mouse models, and a common factor in all except the c-*Mos* knockout is an FVB progenitor background (S1 Table). Intriguingly, when viewed as a group, meiotic abnormalities and parthenogenetic activation are not universal features of these models, but defects in follicle recruitment and/or growth are, although the phenotypic variability among studies is considerable.

Follicle abnormalities alone, however, are not sufficient for the induction of OTs. Impaired follicle development occurs in both *Foxo3a* missense mutation and null mice, but OTs only develop in the missense mutation line [25]. Similarly, teratomas develop in mice overexpressing the anti-apoptotic factor *Bcl2* but not in mice null for *Bax*, a pro-apoptotic factor analogous to *Bcl2* [39,40]. With a single exception, all studies that have examined the oocyte report increased pathenogenetic activation. In this regard, the *Rb1-cKO* mouse studied here and the *Tgkd* transgenic reported by Balakrishnan and Chaillet are notable exceptions, since neither meiotic defects nor an increased level of parthenogenetic activation is evident in either model [28]. Instead, in both models follicle defects appear to drive the tumor phenotype. In *Tgkd* transgenics, an ovulation defect appears to result in the entrapment of mature eggs in an un-ruptured, luteinized follicle. In our model, loss of Rb1 function appears to lead to an uncoupling of coordinated oocyte and granulosa growth.

On the basis of our findings and those reported from analyzing other OT models, we conclude that folliculogenesis defects and a permissive genetic background are sufficient to drive OT development, even in the absence of meiotic defects that enhance parthenogenetic activation. That is, in both *Rb1-cKO* and *Tgkd* transgenic mice the follicle defect is apparently the driver of the OT phenotype. Importantly, however, follicle defects are also a feature of OT models with an increased incidence of parthenogenetic activation. Indeed, our study is the third to note an association between increased follicle recruitment and OT formation [25,41]. In the case of the constitutively active *Fshr* gene, although the effect of activating this receptor on the initial recruitment of follicles into the growing pool in the immature ovary has not, to our knowledge, been examined, activation of this receptor is the basis of many follicle stimulation techniques. Thus, the larger litter sizes in these females and the fact that their supply of follicles is depleted early suggests both enhanced recruitment and the rescue of a population of growing follicles that normally would not be ovulated. However, unregulated premature follicle recruitment is also a feature of mice with a *Foxo3a* missense mutation [25]. Indeed, in all studies that have examined OT formation, defects in growing follicles have been noted, although on initial examination, there is no commonality. This prompted us to examine the results of previous studies more closely and, in all studies but one, a case can be made for dysregulation between the oocyte and somatic components of the follicle. Intriguingly, in several models, enlarged oocytes are reported in primordial follicles suggesting growth asynchrony even at the very earliest stages of follicle growth [25,28,40,41]. Although follicle growth is also dysregulated in *Rb1-cKO* mice, the phenotype is slightly different because more follicles are recruited in the first wave of folliculogenesis. Our data also suggest an uncoupling of oocyte and somatic cell growth; this phenotype, however, does not become evident until follicles reach the secondary stage.

Teratomas appear to arise from a small number of oocytes that undergo premature activation. We discovered a low level of parthenogenetic activation in culture and a low number of abnormal oocytes in *Rb1-cKO* ovaries. Furthermore, teratoma formation was found to always be unilateral in *Rb1* germ cell deficient mice. These findings suggest that most *Rb1*-deficient oocytes resist teratoma formation. Although these phenotypes seem odd, they are in line with observations from human cases of hereditary retinoblastoma disease. In several instances, retinoblastoma disease occurs in one eye only, even when *Rb1* is mutated in all cells of the body [42]. Results of a previous study found that the incidence of retinoblastoma occurs in only 36% of patients with certain *Rb1* mutations [43]. Collectively, these findings suggest the existence of a safeguard mechanism that protects against Rb1-deficiency in some, but not all, cells. Furthermore, only 30% of *c-Mos* null females develop ovarian teratoma between 4 to 8 months of age [44], suggesting a similar undiscovered machinery also exists in other OT models.

The source of ovarian teratomas due to mutations could be caused by defects in either somatic cells or germ cells. Indeed, loss-of-function mutations in the *Rb1* gene have been reported in non-teratoma human ovarian tumors that are somatic cell or germ cell in origin [22,45]. However, whether Rb1 deficiency in granulosa cells or germ cells leads to teratoma formation has been unclear. Previous studies in mice revealed that premature exhaustion of the follicle pool occurs following conditional inactivation of *Rb1* in granulosa cells specifically, but formation of ovarian teratomas was not reported [46]. Thus, the origin of ovarian teratomas in the current study is due to defects in the oocyte specifically.

In conclusion, it is clear that neither meiotic abnormalities nor a propensity for parthenogenetic activation are essential for OT formation. Importantly, the fact that *Rb1-cKO* females recapitulate some of the follicle defects reported in several previous studies has allowed us to define an important cause of OT formation. We postulate that dysregulation between growth of the oocyte and the somatic component of the follicle results in an oocyte that attains meiotic competence in a follicle with a granulosa cell compartment that is insufficient to maintain meiotic arrest. This results in the premature resumption of meiosis in a secondary follicle and, with this scenario, even the very low basal level of spontaneous activation becomes sufficient to induce low-level OT formation.

## Materials and Methods

### Animals

All animal procedures were conducted in accordance with the Guide for the Care and Use of Laboratory Animals and approved by the Washington State University Institutional Animal Care and Use Committee (IACUC), which is fully accredited by the American Association for Accreditation of Laboratory Animal Care. *Rb1* floxed (*Rb1* ^*fl/fl*^) mice were obtained from the NCI mouse repository (Frederick, MD, USA), and *Ddx4-Cre* and *Blimp1-Cre* transgenic mice were obtained from The Jackson Laboratory (Bar Harbor, ME, USA). All mice were maintained on a mixed 129;FVB genetic background and bred as described previously [47]. Briefly, *Rb1* ^*fl/fl*^ females were mated with *Ddx4-Cre or Blimp1-Cre* males to generate *Ddx4-Cre;Rb1* ^*fl/+*^ or *Blimp1-Cre;Rb1* ^*fl/+*^ mice. Young (<3 months of age) *Ddx4-Cre;Rb1* ^*fl/+*^ were crossed with *Rb1* ^*fl/fl*^ or *Rb1* ^*fl/+*^ to generate *Ddx4-Cre*;*Rb1* ^*fl/∆*^, *Ddx4-Cre*;*Rb1* ^*+/∆*^, *Ddx4-Cre*;*Rb1* ^*fl/+*^, *Rb1* ^*fl/∆*^, *Rb1* ^*+/∆*^, *Rb1* ^*fl/fl*^ and *Rb1* ^*fl/+*^ mice. The *Ddx4-Cre*;*Rb1* ^*fl/∆*^ animals were considered *Rb1* germline homozygous knockouts. Control mice were *Rb1* ^*fl/fl*^ and *Rb1* ^*fl/+*^ littermates. *Blimp1-Cre; Rb1* ^*fl/+*^ males were mated with Rb1^fl/*∆*^ to generate *Blimp1-Cre;Rb1* ^*fl/∆*^ and control animals. Wild-type 129;FVB males were used for assessing fertility status.

### Immunohistochemical staining of ovarian cross-sections

Ovaries were fixed in 4% PFA and embedded in paraffin. The presence of Rb1, phospho-Rb1, and Ddx4 protein was detected by immunohistochemistry. Briefly, antigen retrieval was achieved by incubating sections in boiling Na Citrate buffer. Sections were then incubated with 10% normal donkey or goat serum for 1 h at room temperature to block for non-specific antibody binding followed by incubation with primary antibodies (S2 Table) overnight at 4°C. Sections were then washed in PBS and incubated with secondary antibodies (S2 Table) for 2 h at room temperature. For detection of immunofluorescent signal, slides were mounted with Prolong gold anti-fade reagent containing DAPI (Life Technologies, CA, USA). For colorimetric signal, slides were treated with an endogenous Biotin-Blocking Kit (Life Technologies, CA, USA), followed by detection with a DAB staining kit (Vector Laboratories, CA, USA). Digital images were captured with a DP72 microscope camera and CellSense acquisition software (Olympus, PA, USA).

### Isolation and culture of oocytes

Female *Rb1-cKO* mice at 22 to 25 days of age were injected with 5 IU equine chorionic gonadotropin (PMSG, The National Hormone and Peptide Program [NHPP], CA, USA). Germinal vesicle (GV) stage oocytes were collected 44–45 h after PMSG injection and cultured. GV breakdown (GVBD) and polar body (PB) extrusion were assessed after 2 and 17 h in culture, respectively. To test for parthenogenetic activation, oocytes were cultured for 48 h and the percentage of 2-cell embryos was quantified based on morphology. In initial studies, we used Waymouth’s MB752/1 medium (Gibco, Life Technologies, CA, USA) supplemented with 10% fetal calf serum and 0.23 mM sodium pyruvate as described previously [48]. The culture condition supported oocyte maturation and parthenogenetic activation because more than 50% of control oocytes treated with 7% ethanol for 5 min developed to 2-cell embryos. When no evidence of parthenogenesis was observed, we conducted a second series of experiments replicating a previously described culture protocol [27] in which GV oocytes were collected and cultured in Minimal Essential Medium (MEM) with Earle’s balanced salt solution (Sigma, MO, USA) supplemented with essential amino acids (Sigma, MO, USA), Penicillin and Streptomycin (Gibco, Life Technologies, CA, USA), 0.01 mM Ethylenediaminetetraacetic acid tetrasodium salt hydrate (Tetrasodium EDTA, Sigma, USA), 0.23 mM pyruvic acid (Sigma, USA) and 3% bovine serum albumin (BSA, Sigma, USA). GV breakdown (GVBD), polar body (PB) extrusion and parthenogenetic activation were assessed as described previously [27].

### Analysis of meiotic spindle formation

MII oocytes were collected from female mice at 25 to 27 days of age. After PMSG injections, mice were injected with 5 IU hCG (NHPP, CA, USA) and oocytes collected into PBS 15 h later. Oocytes were fixed in a microtubule-stabilizing buffer and stained for α-tubulin as described previously [49]. After staining, slides were mounted with Prolong Gold anti-fade reagent containing DAPI (Life Technologies, CA, USA) and chromosome alignment was examined using fluorescent microscopy.

### Statistics

All quantitative data are presented as mean±SEM for at least 3 biological replicates. Differences between means were determined using the general linear model one-way ANOVA function of SAS software (SAS Institute, USA). Multiple comparison analysis was conducted using Tukey’s posthoc test. Differences between means were considered significant at P<0.05.

## Supporting Information

S1 Fig Rb1 phosphorylation in granulosa cells and oocytes. Representative images of immunostaining for the phosphorylated (pSer780, pSer795 and pSer807/811) forms of Rb1 in cross-sections from wild-type mice. Normal IgG was used as a negative control. Scale bar = 50μm.
(TIF)

S2 Fig Unilateral ovarian teratoma formation in mice with conditional inactivation of *Rb1* in oocytes. (A) Representative image of control mice without tumors and *Rb1-cKO* mice possessing tumors in one ovary with the contralateral ovary being normal. (B) Gross morphology of a dissected ovarian tumor from an *Rb1-cKO* mouse. Scale bar = 1cm.
(TIF)

S3 Fig Detection of *Ddx4-Cre* activity in oocytes. Representative images of immunostaining with an antibody to RFP in ovarian cross-sections from *Ddx4-Cre;RFP* ^*floxed-stop-floxed*^ and control mice. Scale bar = 50μm.
(TIF)

S4 Fig Ovarian tumor formation in *Blimp1-Cre;Rb1* ^fl/∆^ mice. (A) Representative images of control and abnormal ovaries from *Blimp1-Cre;Rb1* ^*fl/∆*^ mice. Scale bar = 1 cm. (B) Representative images of hematoxylin and eosin (H&E) stained cross-sections from abnormal ovaries of *Blimp1-Cre;Rb1* ^*fl/∆*^ mice.
(TIF)

S5 Fig Ovarian tumors in mice with conditional inactivation of Rb1 contain cells that express markers of pluripotency. (A) Representative images of hematoxylin and eosin (H&E) stained cross-sections from normal ovaries of adult (2 months of age) *Rb1-cKO* mice. (B & C) Representative images of H&E stained cross-sections from abnormal ovaries of adult *Rb1-cKO* mice. *indicates ovarian cysts. (D) Representative images of immunostaining for the pluripotency markers Oct4 and Nanog in cross-sections from normal and cystic ovaries of adult *Rb1-cKO* mice.
(TIF)

S6 Fig Germline deficiency of Rb1 does not impact primordial follicle number. (A) Representative images of hematoxylin and eosin (H&E) stained cross-sections from ovaries of control and *Rb1-cKO* mice at postnatal day (PD) 2. (B) Quantitative comparison of the number of primordial follicles in ovarian cross-sections from control and *Rb1-cKO* mice at PD 2.
(TIF)

S7 Fig Germline deficiency of Rb1 leads to impaired follicle growth. (A) Representative images of hematoxylin and eosin (H&E) stained cross-sections from ovaries of control and *Rb1-cKO* mice at postnatal day (PD) 45. (B) Quantitative comparison of the number of secondary and preantral follicles in ovarian cross-sections from control and *Rb1-cKO* mice at PD 45. (C) Representative images of H&E stained cross-section from ovaries of control and *Rb1-cKO* mice at PD 150–180. (D) Quantitative comparison of the number of secondary and preantral follicles in cross-sections of ovaries from control and *Rb1-cKO* mice at PD 150–180. (E) Representative images of H&E stained cross-sections from ovaries of control and *Rb1-cKO* mice at PD 240. (F) Quantitative comparison of the number of secondary and preantral follicles in cross-sections of ovaries from control and *Rb1-cKO* mice at PD 240. All quantitative data are presented as the mean±SEM for at least 3 different mice. Scale bars = 50 μm.
(TIF)

S8 Fig Ovarian teratomas in mice with germline conditional inactivation of *Rb1* likely arise from post-meiotic oocytes. (A) Representative images of immunostaining for the pluripotency markers Oct4 and Nanog in cross-sections of ovaries from *Rb1-cKO* mice at postnatal day PD 21. Scale bars = 50μm. (B) Representative images of agarose gels from genotypic analysis of teratomas and host somatic tissues for the *Rb1* floxed allele (280 bp), recombined allele (260 bp) and wild type allele (235 bp).
(TIF)

S1 Table List of ovarian teratoma (OT) mouse models. (DOCX)

S2 Table List of primary and secondary antibodies used for immunostaining. (DOCX)
