# Supplementary figures and images for: Inactivation of Retinoblastoma Protein (Rb1) in the Oocyte: Evidence That Dysregulated Follicle Growth Drives Ovarian Teratoma Formation in Mice
Source: PLoS Genet. 2015 Jul 15;11(7):e1005355. doi: 10.1371/journal.pgen.1005355 (PMC4503754; doi:10.1371/journal.pgen.1005355)

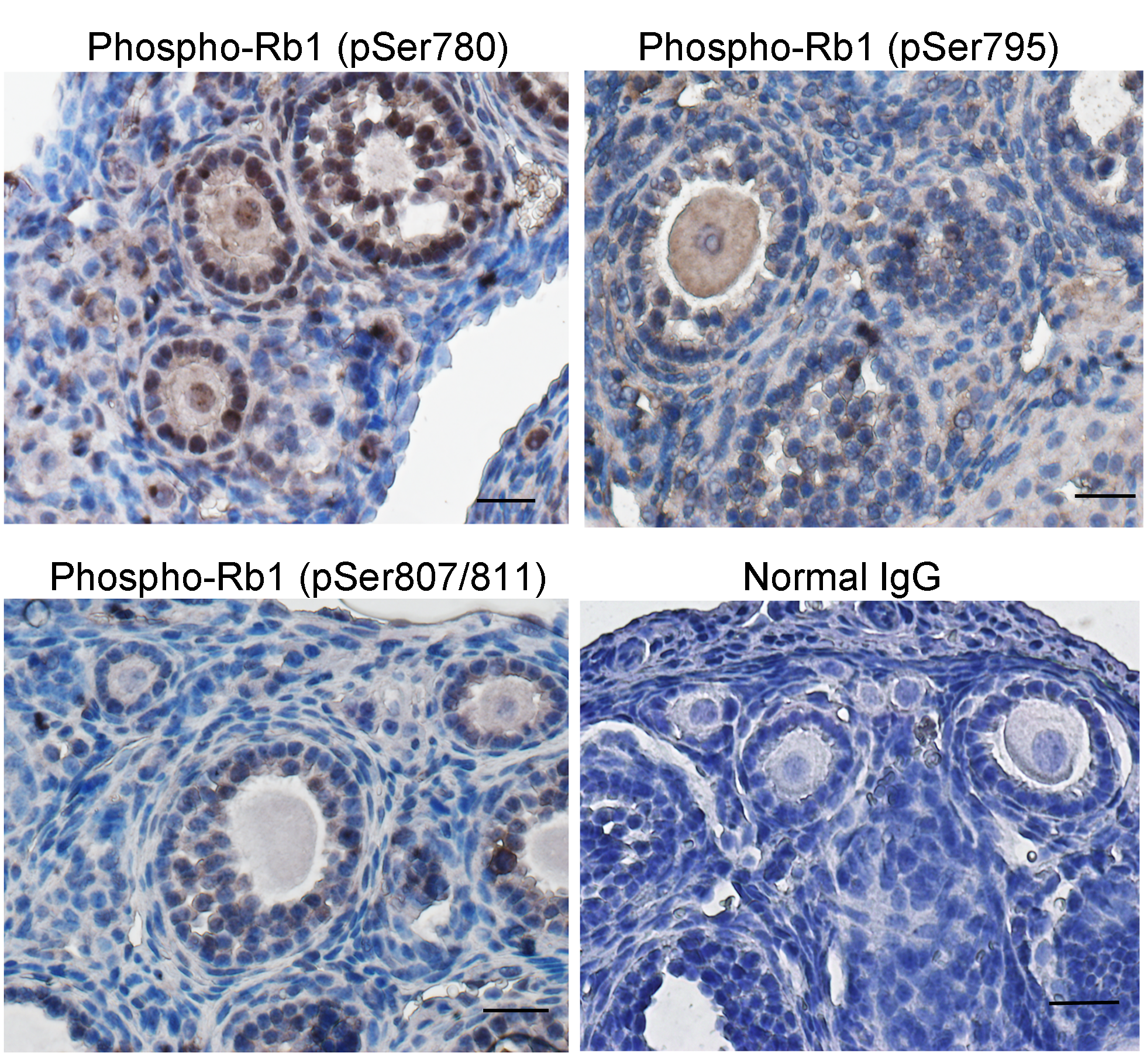

Supplement: S1 Fig — Representative images of immunostaining for the phosphorylated (pSer780, pSer795 and pSer807/811) forms of Rb1 in cross-sections from wild-type mice. Normal IgG was used as a negative control. Scale bar = 50μm. (TIF) [file pgen.1005355.s001.tif]

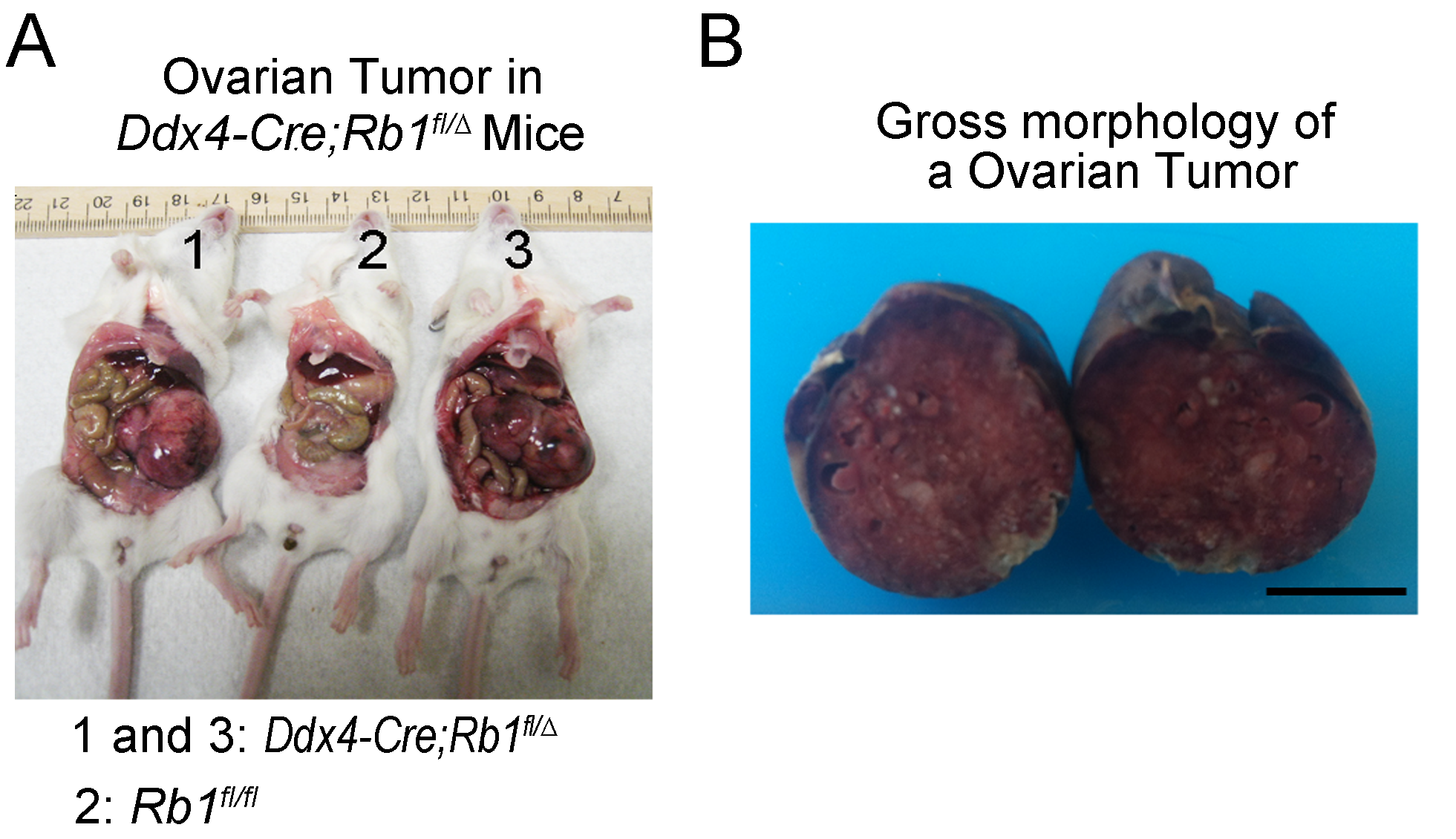

Supplement: S2 Fig — (A) Representative image of control mice without tumors and Rb1-cKO mice possessing tumors in one ovary with the contralateral ovary being normal. (B) Gross morphology of a dissected ovarian tumor from an Rb1-cKO mouse. Scale bar = 1cm. (TIF) [file pgen.1005355.s002.tif]

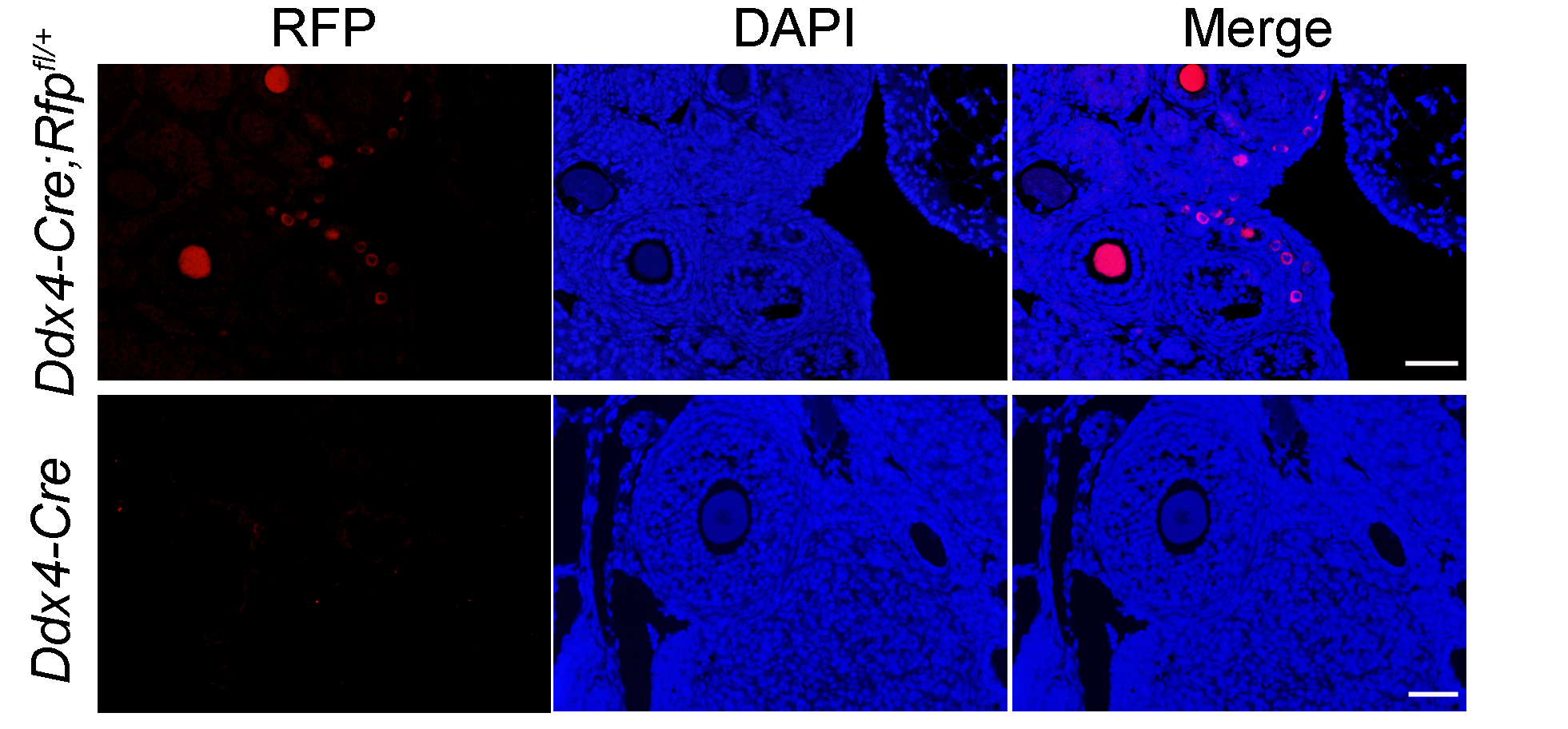

Supplement: S3 Fig — Representative images of immunostaining with an antibody to RFP in ovarian cross-sections from Ddx4-Cre;RFP floxed-stop-floxed and control mice. Scale bar = 50μm. (TIF) [file pgen.1005355.s003.tif]

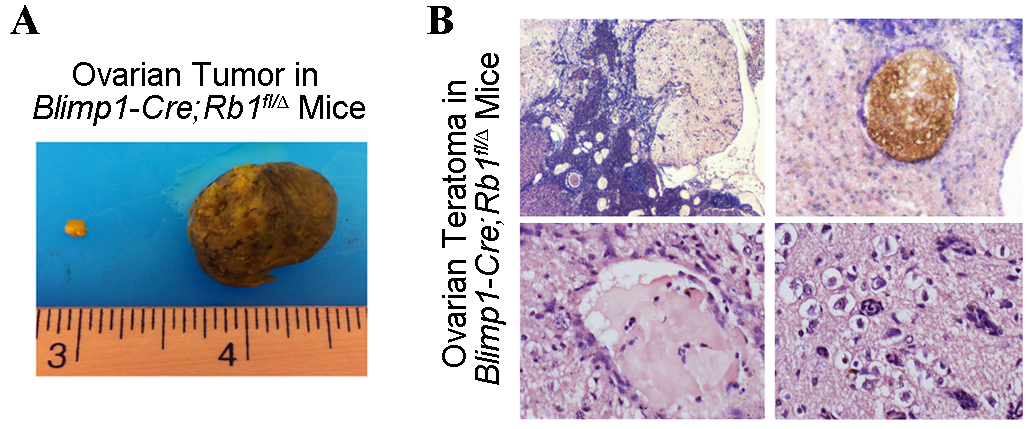

Supplement: S4 Fig — (A) Representative images of control and abnormal ovaries from Blimp1-Cre;Rb1 fl/∆ mice. Scale bar = 1 cm. (B) Representative images of hematoxylin and eosin (H&E) stained cross-sections from abnormal ovaries of Blimp1-Cre;Rb1 fl/∆ mice. (TIF) [file pgen.1005355.s004.tif]

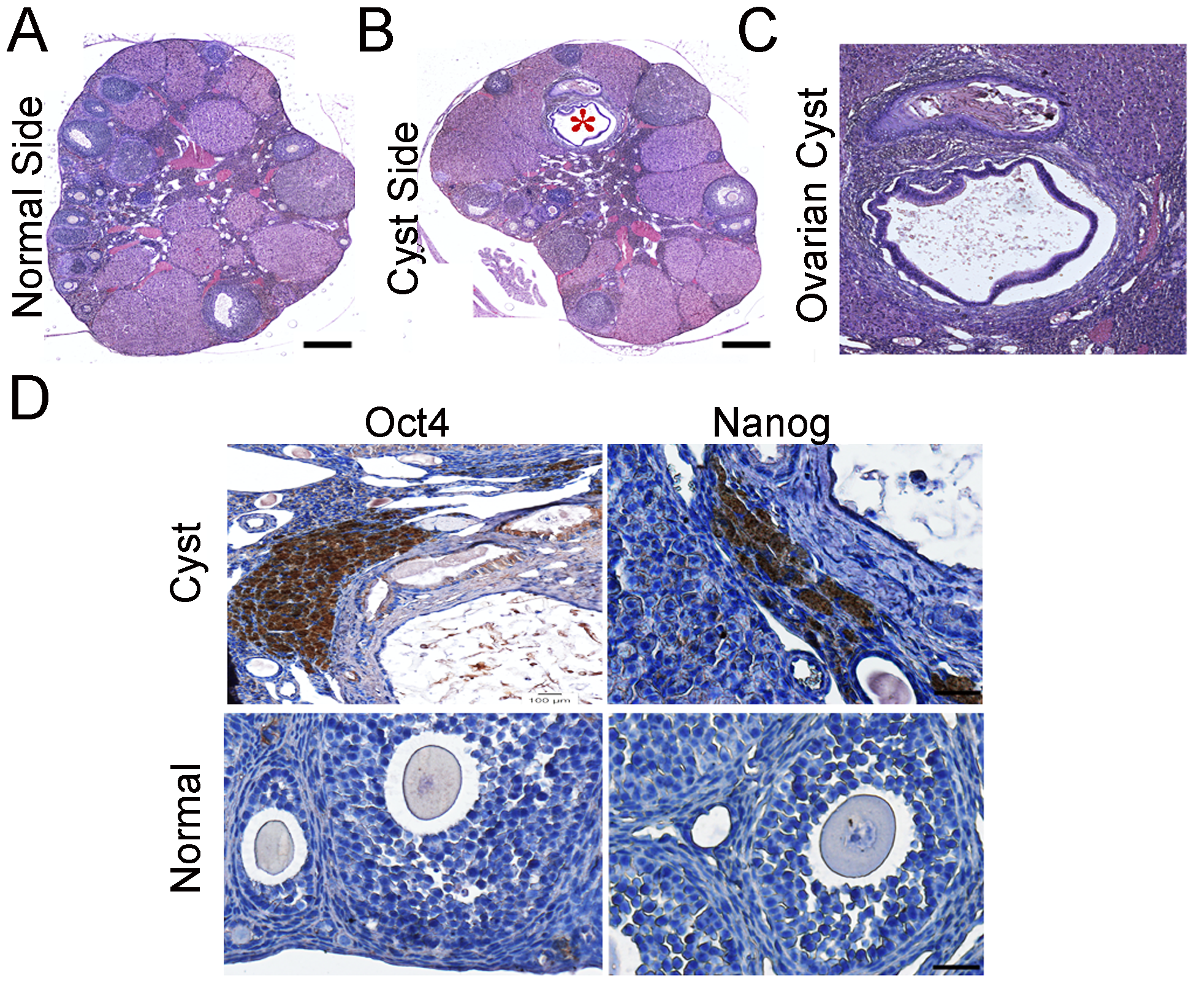

Supplement: S5 Fig — (A) Representative images of hematoxylin and eosin (H&E) stained cross-sections from normal ovaries of adult (2 months of age) Rb1-cKO mice. (B & C) Representative images of H&E stained cross-sections from abnormal ovaries of adult Rb1-cKO mice. *indicates ovarian cysts. (D) Representative images of immunostaining for the pluripotency markers Oct4 and Nanog in cross-sections from normal and cystic ovaries of adult Rb1-cKO mice. (TIF) [file pgen.1005355.s005.tif]

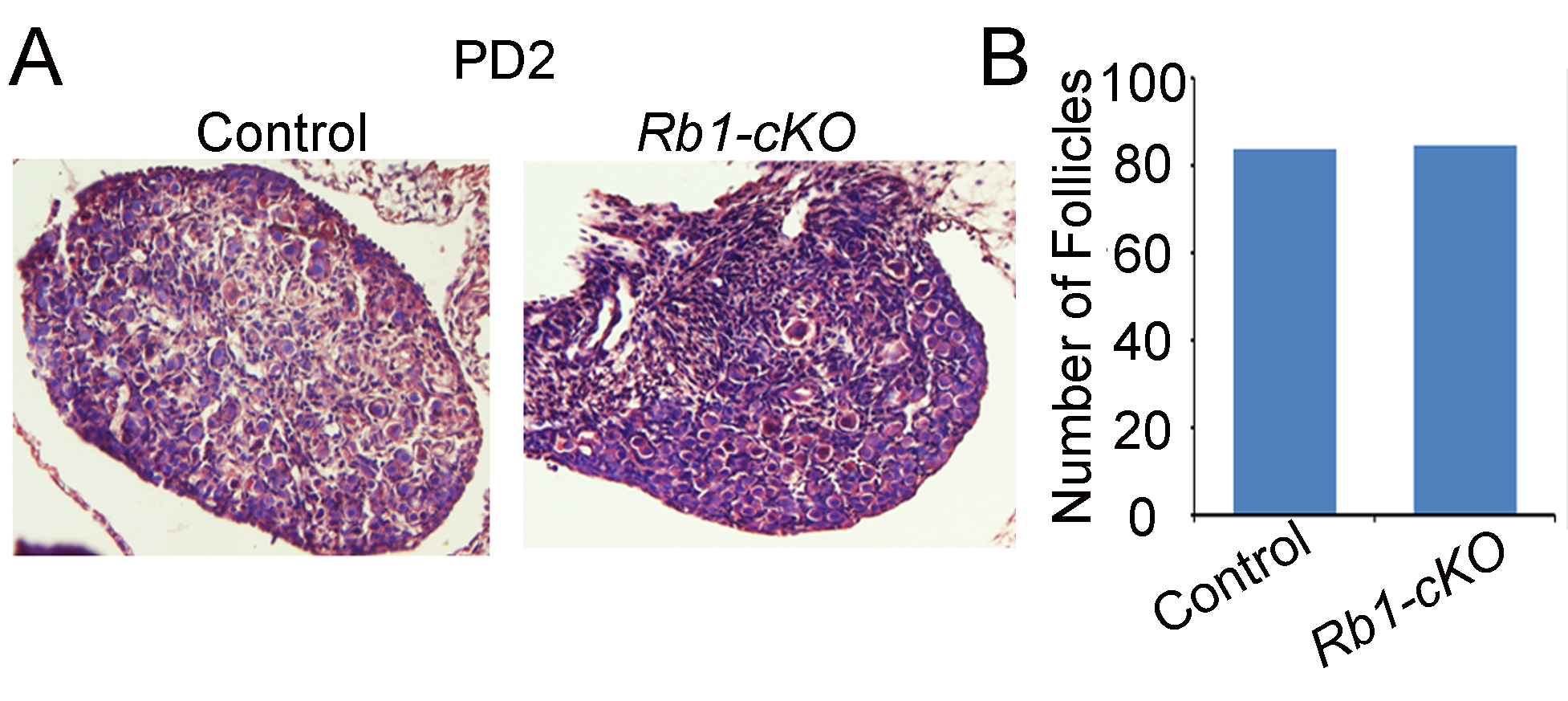

Supplement: S6 Fig — (A) Representative images of hematoxylin and eosin (H&E) stained cross-sections from ovaries of control and Rb1-cKO mice at postnatal day (PD) 2. (B) Quantitative comparison of the number of primordial follicles in ovarian cross-sections from control and Rb1-cKO mice at PD 2. (TIF) [file pgen.1005355.s006.tif]

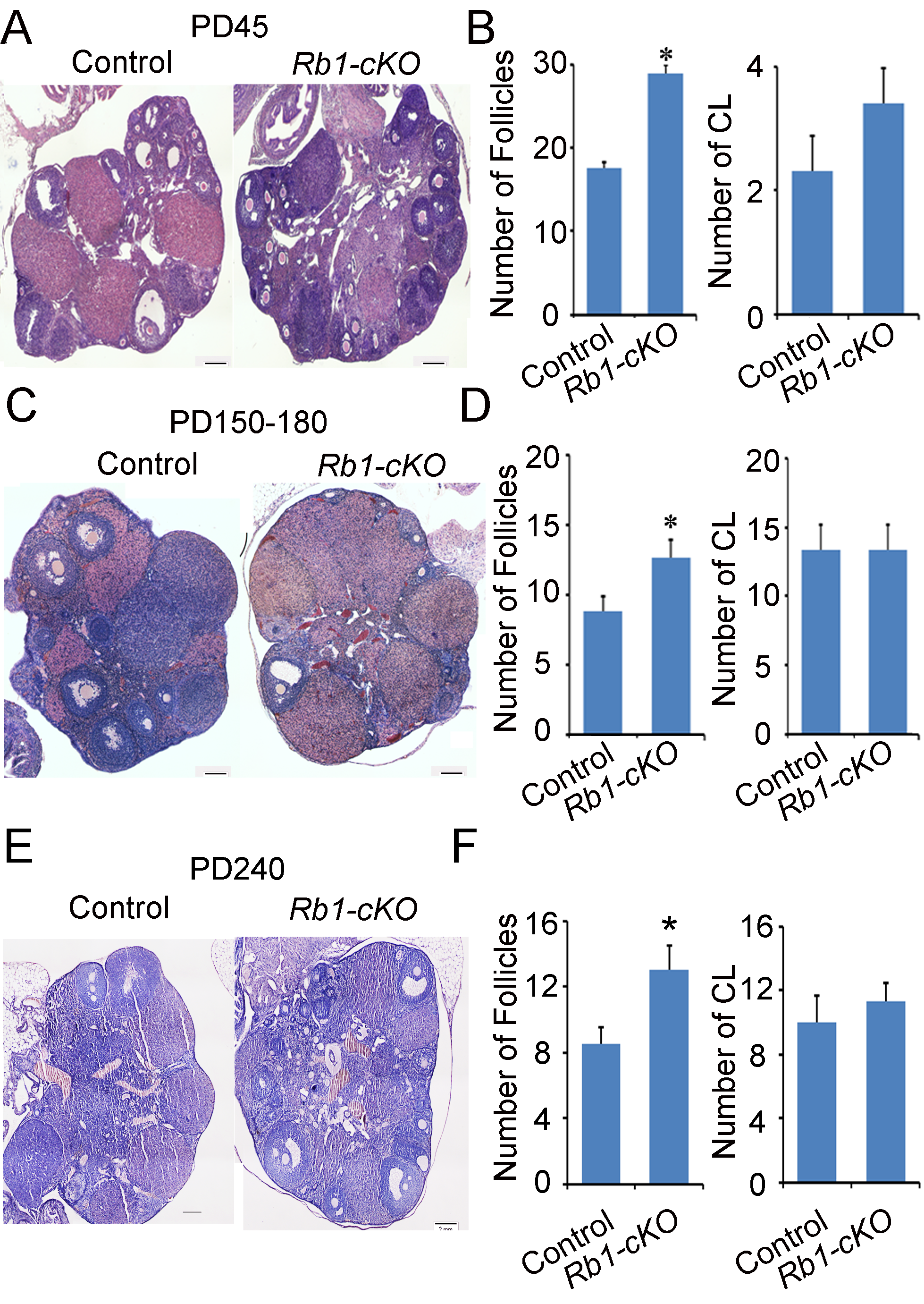

Supplement: S7 Fig — (A) Representative images of hematoxylin and eosin (H&E) stained cross-sections from ovaries of control and Rb1-cKO mice at postnatal day (PD) 45. (B) Quantitative comparison of the number of secondary and preantral follicles in ovarian cross-sections from control and Rb1-cKO mice at PD 45. (C) Representative images of H&E stained cross-section from ovaries of control and Rb1-cKO mice at PD 150–180. (D) Quantitative comparison of the number of secondary and preantral follicles in cross-sections of ovaries from control and Rb1-cKO mice at PD 150–180. (E) Representative images of H&E stained cross-sections from ovaries of control and Rb1-cKO mice at PD 240. (F) Quantitative comparison of the number of secondary and preantral follicles in cross-sections of ovaries from control and Rb1-cKO mice at PD 240. All quantitative data are presented as the mean±SEM for at least 3 different mice. Scale bars = 50 μm. (TIF) [file pgen.1005355.s007.tif]

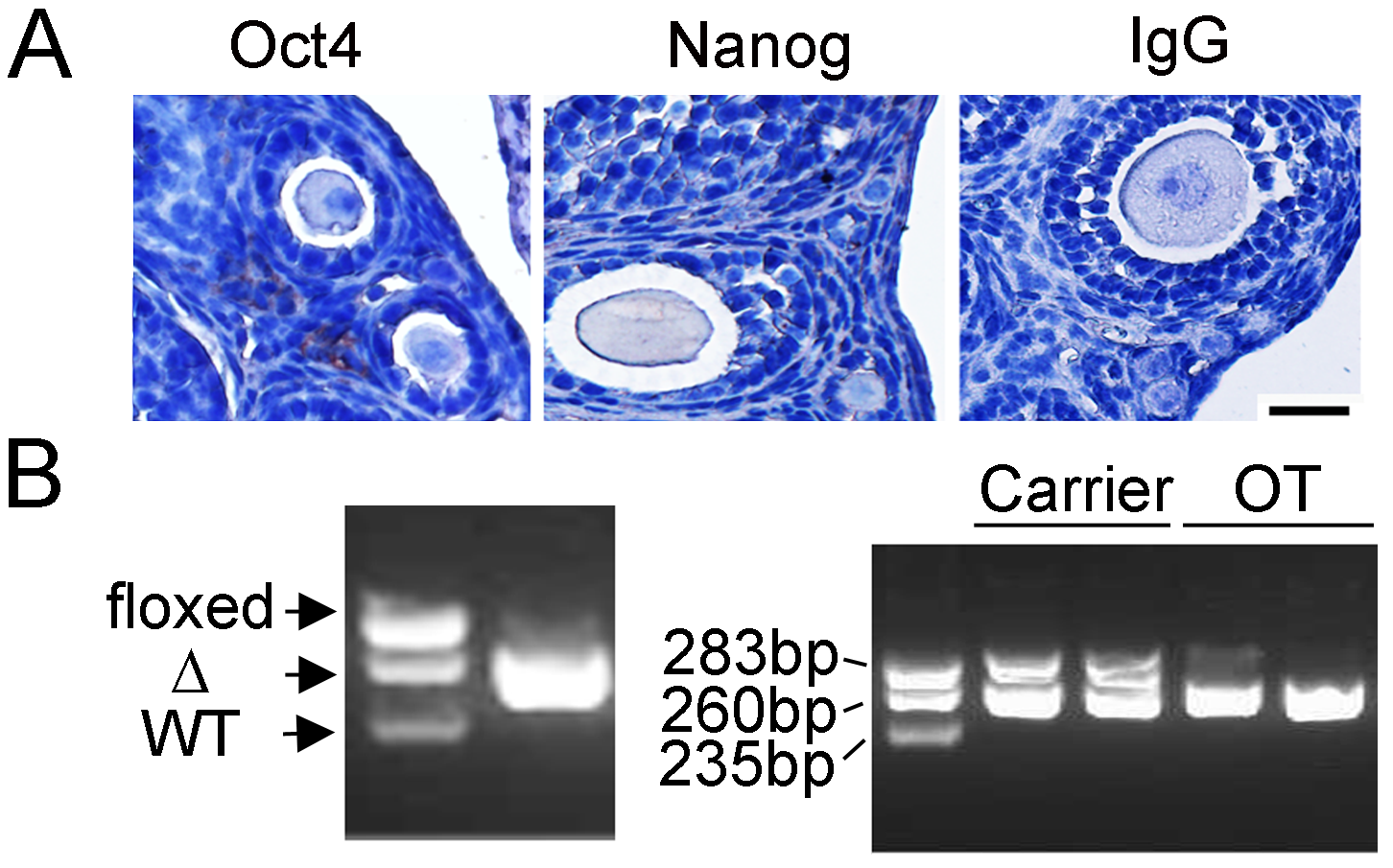

Supplement: S8 Fig — (A) Representative images of immunostaining for the pluripotency markers Oct4 and Nanog in cross-sections of ovaries from Rb1-cKO mice at postnatal day PD 21. Scale bars = 50μm. (B) Representative images of agarose gels from genotypic analysis of teratomas and host somatic tissues for the Rb1 floxed allele (280 bp), recombined allele (260 bp) and wild type allele (235 bp). (TIF) [file pgen.1005355.s008.tif]
