# Supplementary material for: Inactivation of Retinoblastoma Protein (Rb1) in the Oocyte: Evidence That Dysregulated Follicle Growth Drives Ovarian Teratoma Formation in Mice
Source: PLoS Genet. 2015 Jul 15;11(7):e1005355. doi: 10.1371/journal.pgen.1005355 (PMC4503754; doi:10.1371/journal.pgen.1005355)
Supplement: S1 Table — (DOCX) [file pgen.1005355.s009.docx]

**Supplemental Table 1. List of ovarian teratoma (OT) mouse models.**

| Model | FVB Progenitor | OT Incidence | Meiotic abnormalities | Patherno-genetic activation | Follicle defects | Reference |
| --- | --- | --- | --- | --- | --- | --- |
| LT/Sv stain | No | 28% | MI arrest | Yes | Growth asynchrony | ([1](#_ENREF_1), [2](#_ENREF_2)) |
| *Mos* knockout | No | 30% | MII arrest | Yes | Oocyte activation in secondary follicles | ([3](#_ENREF_3)) |
| *Bcl-2* overexpression in the ovary | Yes | 20% | ND | Yes | Increased granulosa cell number | ([4](#_ENREF_4)) |
| *Gata4* global knockdown | Yes | 10% | ND | ND | ND | ([5](#_ENREF_5)) |
| *Fshr* constitutive activation | Yes | 20% | ND | ND | Increased follicle recruitment | ([6](#_ENREF_6)) |
| *Foxo3a* missense mutation | Yes | 16% | MI arrest | No | Growth asynchrony | ([7](#_ENREF_7)) |
| *Tgkd* transgenic insertion | Yes | 15-20% | No | No | Growth asynchrony | ([8](#_ENREF_8)) |
| *Rb1* germline conditional knockout | Yes | 28% | No | No | Growth asynchrony |  |

Abbreviations:

ND: not determined;

OT: ovarian teratoma;

M: metaphase

1. Eppig JJ (1978) Granulosa cell deficient follicles: occurrence, structure, and relationship to ovarian teratocarcinogenesis in strain LT/Sv mice. (Translated from eng) *Differentiation* 12(2):111-120 (in eng).

2. Eppig JJ, Wigglesworth K, Varnum DS, & Nadeau JH (1996) Genetic regulation of traits essential for spontaneous ovarian teratocarcinogenesis in strain LT/Sv mice: aberrant meiotic cell cycle, oocyte activation, and parthenogenetic development. (Translated from eng) *Cancer Res* 56(21):5047-5054 (in eng).

3. Hirao Y & Eppig JJ (1997) Parthenogenetic development of Mos-deficient mouse oocytes. (Translated from eng) *Mol Reprod Dev* 48(3):391-396 (in eng).

4. Hsu SY, Lai RJ, Finegold M, & Hsueh AJ (1996) Targeted overexpression of Bcl-2 in ovaries of transgenic mice leads to decreased follicle apoptosis, enhanced folliculogenesis, and increased germ cell tumorigenesis. (Translated from eng) *Endocrinology* 137(11):4837-4843 (in eng).

5. Thurisch B*, et al.* (2009) Transgenic mice expressing small interfering RNA against Gata4 point to a crucial role of Gata4 in the heart and gonads. (Translated from eng) *J Mol Endocrinol* 43(4):157-169 (in eng).

6. Peltoketo H*, et al.* (2010) Female mice expressing constitutively active mutants of FSH receptor present with a phenotype of premature follicle depletion and estrogen excess. (Translated from eng) *Endocrinology* 151(4):1872-1883 (in eng).

7. Youngson NA*, et al.* (2011) A missense mutation in the transcription factor Foxo3a causes teratomas and oocyte abnormalities in mice. (Translated from eng) *Mamm Genome* 22(3-4):235-248 (in eng).

8. Balakrishnan A & Chaillet JR (2013) Role of the inositol polyphosphate-4-phosphatase type II Inpp4b in the generation of ovarian teratomas. (Translated from eng) *Dev Biol* 373(1):118-129 (in eng).
