# Supplementary material for: Inactivation of Retinoblastoma Protein (Rb1) in the Oocyte: Evidence That Dysregulated Follicle Growth Drives Ovarian Teratoma Formation in Mice
Source: PLoS Genet. 2015 Jul 15;11(7):e1005355. doi: 10.1371/journal.pgen.1005355 (PMC4503754; doi:10.1371/journal.pgen.1005355)
Supplement: S2 Table — (DOCX) [file pgen.1005355.s010.docx]

**Supplemental Table 2: Primary and secondary antibodies used for immunostaining.**

| Antibody | Dilution | Source | Reference |
| --- | --- | --- | --- |
| Primary Antibody |  |  |  |
| Rb1 Mouse anti-human IgG | 1:50 | BD Pharmingen | Cat. 554136 |
| Phospho-Rb1(Ser780) Rabbit anti-mouse IgG  Ddx4 Rabbit anti-mouse IgG  Oct4 Rabbit anti-mouse IgG  Nanog Rabbit anti-mouse IgG α-Tubulin Monoclonal mouse IgG | 1:200  1:500  1:200  1:200 | R&D system  Cell Signaling Technology  Abcam  Abcam  Sigma-Aldrich | Cat. 9306  Cat. ab13840  Cat.ab19857  Cat.ab80892  DM1A, T9026 |
| Secondary Antibody |  |  |  |
| Alexa Fluor 546 Donkey anti-mouse IgG | 1:1000 | Invitrogen | Cat.A10036 |
| Alexa Fluor 488 Donkey anti-rabbit IgG  Goat anti-rabbit IgG-B  Goat anti-mouse IgG-B | 1:1000  1:500  1:500 | Invitrogen  Santa Cruz  Santa Cruz | Cat.A11008  Cat. sc-2040  Cat. sc-2072 |
